# Mean reticolocyte hemoglobin content index plays a key role to identify children who are carriers of β-thalassemia

**Published:** 2018-03-31

**Authors:** Paolo Vicinanza, Mariella Vicinanza, Vincenzo Cosimato, Daniela Terracciano, Sergio Cancellario, Angelo Massari, Paolo Danise, Carmine Selleri, Bianca Serio

**Affiliations:** 1 Clinical Pathology, AOU “OO.RR. San Giovanni di Dio Ruggi d’Aragona”, Salerno, Italy; 2 Medical Genetics, Institute for Medical Research, Cambridge, UK; 3 Translational Medical Science, University of Naples “Federico II”, Italy; 4 Hematology Laboratory, Umberto I Hospital, Nocera Inferiore, Italy; 5 Hematology and Bone Marrow Transplant Center, AOU “OO.RR. San Giovanni di Dio Ruggi d’Aragona”, University of Salerno, Italy; 6 Department of Medicine and Surgery, University of Salerno, Baronissi, Italy

**Keywords:** β-Thalassemia, reticulocyte Indices, iron deficiency, DI-BTT

## Abstract

Reticulocyte (r) and red blood cell (RBC) indices provide reliable parameters for screening and monitoring iron deficiency anemia (IDA) patients and β-thalassemia trait (BTT) carriers. The aim of this study is to identify a simple method for use to distinguish β-thalassemia trait carriers from IDA and to evaluate the correlation between BTT genetic mutation and MCV values and new discrimination index for the detection of β-thalassemia trait (DI-BTT).

We analyzed CHr, MCHCr, MCVr, RBC, mean cellular hemoglobin concentration (MCHC) and mean cellular volume (MCV) indices among a pediatric population of IDA patients (n=90), β-thalassemia trait carriers (n=72) and normal controls (NC) (n=131). Furthermore, to distinguish IDA patients from β-thalassemia trait carriers we evaluated clinical utility of new DI for the detection BTTcarriers, using the following polynomial: (RBC × MCHC × 50/MCV)/CHr.

We found that CHr, MCVr and DI-BTT mean values were significantly different between β-thalassemia trait carriers and IDA patients. CHr, MCVr and DI-BTT plotting curves showed exclusive distribution in β-thalassemia trait carriers. Moreover, DI-BTT was very accurate in differentiating β-thalassemia trait carriers from IDA patients. All BTT patients showed a heterozygous mutation of the β-globin gene including CD39, IVS1.110, IVS1.6 and IVS2.745, IVS2.1 and IVS1.1. The highest MCV values were displayed by those carrying the IVS1.6 mutation.

**Conclusions:**

The simultaneous measurement and plotting of CHr and MCVr indices, as well as the DI-BTT allow to distinguish β-thalassemia carriers from IDA patients.

## I. INTRODUCTION

Reticulocyte (r) are immature circulating non-nucleated red cells containing residual ribonucleic acid (RNA) and continuing to synthesize hemoglobin after loss of the nucleus. Several investigators have suggested that reticulocyte indices may differentiate iron deficiency anemia from thalassemia (thal) [1], providing information on bone marrow erythroid activity without expensive or invasive procedures [2]. The potential of reticulocyte indices derives from the short mean life of circulating reticulocyte (24 – 48h), mirroring the late phases of hemoglobin synthesis [3]. Reticulocyte indices such as CHr (mean cellular hemoglobin), MCHCr (mean cellular hemoglobin concentration), MCVr (mean cellular volume) and IRF (immature reticulocyte fraction) provide indirect information about bone marrow erythropoietic activity. In physiological condition, CHr is directly related to MCVr and MCHCr, according to polynomial CHr = MCVr × MCHCr/100 [3]. CHr reflects the degree of effective erythropoiesis of the previous two days and is useful to evaluate functional iron deficiency, to detect the use of blood doping drugs and to monitor iron deficiency anemia (IDA) patients [4, 5]. CHr and MCVr are low not only in IDA but also in thalassemia trait patients [6]. In the last 20 years, molecular studies have clarified the pathogenesis of 80% of β-thalassemia trait (BTT) carriers in the world [7, 8]. Rund et al. have documented that β° and β^+^ thal patients show MCV mean values of 63.1 +/− 3.4 fL and of 69.3 +/− 5.6 fL, respectively [9]. Skarmoutson at al., measured several hematologic parameters including CHr, soluble transferrin receptor (sTfR), HbA_2_% and HbF% in β-thalassemia trait carriers, suggesting that CHr is significantly higher in β° compared to β^+^ thalassemia patients (CHr range: 27÷ 32 pg and 19.5 ÷ 25.3 pg, respectively). Furthermore, they documented that sTfR directly correlated with HbA_2_%, whereas CHr indirectly correlated with HbA_2_%, suggesting that carriers had a variable degree of ineffective erythropoiesis related to the specific β-globin mutation present [10]. The genetic complexity of the beta thalassemia trait is documented by its phenotypic heterogeneity, such as in cases with a: a) normal MCH and increased HbA_2_%; b) reduced MCH and HbA_2_ normal and c) reduced MCH and HbA_2_ increased. We studied reticulocyte indices and their distribution in a microcytic pediatric cohort with iron deficiency anemia or β-thalassemia, also evaluating the potential role of a new discriminator index method for β-thalassemia trait pediatric screening.

## I. PATIENTS AND METHODS

This study is a retrospective analysis of samples from hospitalized patients, outpatients and a group of several schools of the Amalfi coast processed over 5 years in a diagnostic hematology laboratory processing on average 200 hematological samples per day. 293 subjects, age ranging from 7 to 15 years (141 male and 152 female), grouped in β-thalassemia trait carriers (n=72); IDA patients (n=90) and normal controls (NC, n=131) were analysed. β-thalassemia trait carriers were defined according to the following parameters: HbA_2_ > 3.3%, Ferritin ≥ 13 ng/ml, Hb ≤ 11 g/dL, MCV < 80 fL, iron > 36 μg/dL and transferrin saturation > 16.0 %. IDA patients were defined according to HbA_2_ < 3.3 %, Ferritin < 13 ng/ml, Hb ≤ 11 g/dL, MCV< 80 fL, HbF< 2%, iron < 20 μg/dL and transferrin saturation < 16.0 %. Normal controls were defined according to HbA_2_ < 3.3 %, Ferritin ≥ 13 ng/ml, Hb ≥ 11g/dL, MCV ≥ 80 fL, HbF< 2%, iron > 30 μg/dL and transferrin saturation ≥ 17.0 %. Samples from patients exhibiting HbA2 levels ≥ 3.3% were subjected to molecular testing for genetic mutations of the β-globin gene.

This study was conducted according to Good Clinical Practice guidelines and the provisions of the declaration of Helsinki. Protocol approval was obtained from the local ethics committee and all parents of the patients gave informed consent.

### Blood Samples

Blood samples were obtained in the morning and samples for red cell indices were collected into K_2_-EDTA test tubes. Blood counts as well as red cell markers and reticulocyte indices were measured on an ADVIA 120 Hematology System (Siemens Healthcare Diagnostics, Tarrytown, NY, USA), which is able to count reticulocytes by Oxazine 750 RNA staining. For each patient, we obtained RBC, MCHC, MCV, reticulocyte count, CHr, MCVr and MCHCr. HbA_2_% quantification was performed by cation-exchange high performance liquid chromatography using the β-thalassemia Short Program on the Bio-Rad Variant HPLC apparatus (Bio-Rad Laboratories, Hercules, CA USA). Serum ferritin (Ferr) levels were determined with a routine chemoluminescence analyzer (ADVIA Centaur, Siemens Healthcare Diagnostics, Tarrytown, NY, USA). Serum iron and total iron binding capacity (TIBC) were determined by colorimetry (Beckman Coulter). Moreover, transferrin saturation was calculated using the following formula: (serum iron/TIBC) × 100. The criteria to diagnose iron deficiency were the transferrin saturation of < 16.0% and/or the serum ferritin level of less than 13.0 ng/mL. We studied the distribution curves of CHr, MCVr and DI-BTT of IDA and BTT children and calculate a new index for discrimination of β-thalassemia trait (DI-BTT) carriers: DI-BTT = (RBC × MCHC X 50/MCV)/CHr.( In which the MCHC is the mean corpuscular hemoglobin concentration of the red cell).

Molecular biology analyses were carried out using reagents from Vienna Lab Diagnostics GmbH (Gaudenzdorfer Guertel 43–45 A-1120 Vienna, Austria) as follows: after DNA isolation from patients’ blood samples, PCR amplification was carried out using biotinylated primers and hybridization of amplification products to a test strip containing allele-specific oligonucleotide probes immobilized as an array of parallel lines. Bound biotinylated sequences were detected using streptavidin-alkaline phosphatase and color substrates. The array covers 23 mutations, including the most frequent among Italian population.

### Statistical analysis

Results were expressed as means +/− SD. Quantitative parameters (RBC, MCV, MCHC, CHr, MCVr, MCHCr, HbA_2_, Ferritin, DI-BTT, Serum iron, transferrin saturation percentage and TIBC) were compared between the groups by use of a Student’s unpaired t test (Tab.1). The diagnostic performance of MCVr, MCHCr, CHr was analyzed by Receiver Operating Characteristic (ROC) curve analysis. A P-value of < 0.05 was considered statistically significant. Sensitivity, specificity and accuracy of the DI-BTT have been used as indicators of its effectiveness.

## II. RESULTS

### CHr and MCVr show a different distribution curve in β-thalassemia trait patients

**Fig. 1:**
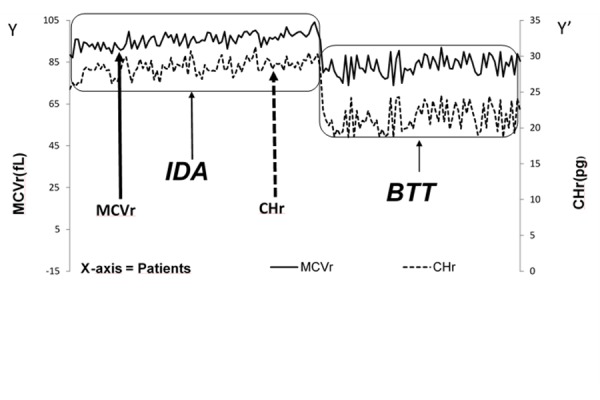
MCVr and CHr values of BTT patients show lower values compared to IDA patients MCVr (Reticulocyte Mean Cellular Volume), CHr (Reticulocyte Mean contente Haemoglobin) in Iron deficient anemia (IDA) and trait β thalassemia carriers (BTT). MCVr (left axis) and CHr (right axis) had a different distribution in BTT patients compared to IDA patients

The mean values of r indices in NC (CHr = 29.44 +/− 1.55 pg; MCVr = 96.31 +/− 3.26 fL; MCHCr = 30.65 +7-0.95 g/dL) and IDA (CHr = 28.29 +/− 1.42 pg; MCVr = 95.57 +7-3.34 fL; MCHCr = 29.68 +/− 1.31 g/dL) children were very similar. Plotting the values of CHr and MCVr indices on axis y/y’, we identified a different distribution between β-thalassemia trait carriers and IDA children (Fig.1).

In NC (data not shown) and IDA patients, we found a similar distribution of the r indices: CHr and MCVr. In β-thalassemia trait carriers, r parameters were significantly different (CHr = 21.44 +/− 1.55 pg; MCVr = 82.06 +/− 5.49 fL; MCHCr =25.74 +/− 1.73 g/dL as well as their distribution (MCVr on the top and CHr below) compared to IDA patients (Fig.1).

This significantly different of r parameters between β-thalassemia trait and IDA children was also confirmed by a Student’s unpaired t test (p < 0.001). This significantly different distribution of r parameters between BTT and IDA children was also confirmed by ROC curve (p < 0.001). The ROC curve analysis showed the largest area under the curve (AUC) comparing BTT and IDA for CHr values (AUC = 0.98; p <0.001), and DI-BTT (AUC = 0.97; p < 0.001), and when comparing NC and BTT for CHr (AUC = 0.99; p < 0.001) and MCHCr (AUC = 0.92; p < 0.001) values. The sensitivity, specificity and accuracy of the DI-BTT are 96 %, 97 % and 96.4 % respectively. In NC, IDA and β-thalassemia trait carriers DI-BTT were 3.43 +/− 0.39, 3.71 +/− 0.39 and 6.93 +/− 1.64, respectively with all p < 0.001 (Tab.1).

All BTT patients showed a heterozygous mutation of the β-globin gene including CD39 mutation in 54%, IVS1.110 in 21%, IVS1.6 and IVS2.745 in 13%, IVS2.1 in 9,84% and IVS1.1 in 6,56% of patients(Tab. 2); IDA and NC patients were conversely wild type in both alleles of the β-globin gene.

**Fig. 2:**
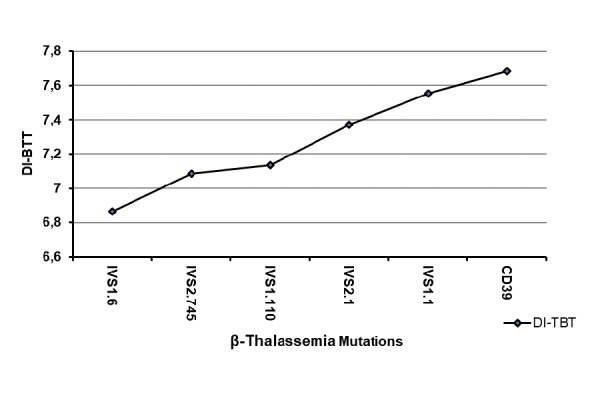
Correlation between discriminant index β Thalassemia Trait (DI-BTT) (y-axis) and six β thalassemia mutations (IVS1.6; IVS2.745; IVS1.110; IVS2.1; IVS1.1 and CD39) more frequent in the pediatric population of Salerno (Italy) (x-axis).

**Fig. 3:**
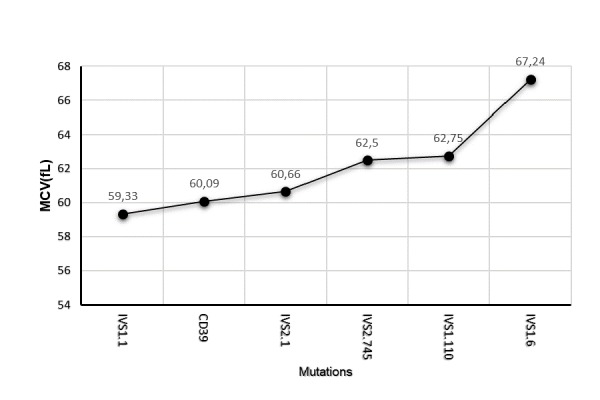
Correlations between mean corpuscolar volume (MCV) and β-thalassemia trait mutations

Interestingly the DI-BTT showed increasing values according to the underlying mutations in the following order IVS1.6<IVS2.745<IVS1.110< IVS2.1<IVS1.1<CD39 (Fig. 2). Moreover, patients carrying the IVS1.1, CD39 and IVS2.1 mutations displayed similar MCV values which were lower when compared to those of patients carrying the IVS2.745 and IVS1.110 mutations. The highest MCV values were displayed by those carrying the IVS1.6 mutations (Fig. 3).

### CHr index combined with red blood cell parameters identify β-thalassemia trait

**Fig. 4:**
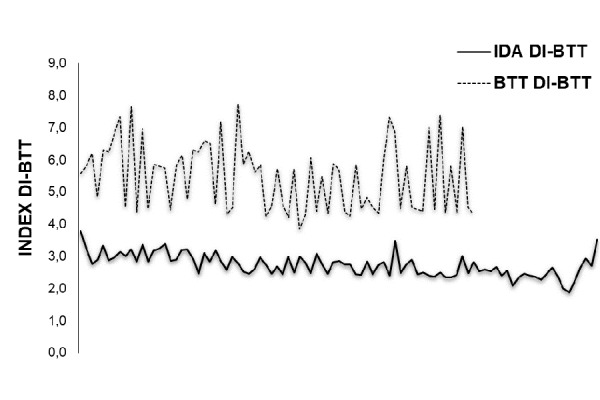
Discriminant index β-Thalassemia Trait (DI-BTT) (DI-BTT) of BTT has higher distribution curve compared to Iron Deficient Anemia (IDA).

Combining red cell parameters such as the number of RBC, CHCM and MCV into the following formula (RBC × MCHC × 50)/MCV and dividing the obtained result for the CHr index, we have identified a new discriminant index (DI) useful for the detection of β-thalassemia trait carriers (DI-BTT). Thus, using the above formula, i.e.(RBC × MCHC × 50/MCV)/CHr, to calculate the DI-BTT we were able to show that while DI-BTT had similar value in NC (3.43 +/− 0.39) and IDA (3.71 +/− 0.39) patients, in β-thalassemia trait children, DI-BTT value was highest (6.93 +/− 1.64), as it can be seen from the curves of distribution of the DI-BTT ( Fig. 4). The differences in DI-BTT between the three groups were statistically significant (p < 0.001) (Tab. 3).

## III. DISCUSSION

The main features of thalassemia syndromes as well as of iron deficiency anemia are microcytosis and hypochromia due to reduced hemoglobinization of RBCs. β-thalassemia trait shows a significant prevalence in the Mediterranean, Middle East, Transcaucasus, Central Asia, Indian subcontinent, and Far East. It is also common in African populations. The highest incidences are reported in Cyprus (14%), Sardinia (12%), and Southeast Asia (11%). However, β-thalassemia trait due to population migration is now also common In Northern Europe, North and South America, in the Caribbean, and in Australia. Since this disease has a very strong socio-economic impact and, diagnosis is usually based on expensive tests, there is a need for rapid and low-cost screening tests allowing the correct diagnosis and the most appropriate management. We have investigated whether the distribution curves of CHr, MCVr indices as well as a new discrimination index, using RBC parameters such as number of RBC, CHCM and MCV values as well as the CHr index, were useful to quickly distinguish IDA patients from β-thalassemia trait carriers. The evaluation of the CHr and MCVr indices showed a different distribution in BTT patients that clearly differed from that in IDA patients and healthy children as in BTT subjects the curves of r indices were lower and more spaced compared to IDA patients (Fig.1). This unique distribution of MCVr and CHr curve indices distinguishing IDA patients from β-thalassemia trait carriers is likely to be related to the more marked microcytosis in β-thalassemia trait carriers compared to IDA patients, but mainly to the deepest hemoglobin genesis deficit as well as to the ineffective erythropoiesis of β-thalassemia trait carriers compared to IDA patients. Various discrimination indices (DIs) for the β-thalassemia trait screening have been proposed but [11, 12] unfortunately, most of them are not easily calculable on all automated analyzers [13]. We developed a unique discrimination index (DI) for β-thalassemia trait screening (DI-BTT), calculable by all instruments using the following formula: (RBC × MCHC × 50/MCV)/CHr. The evaluation of this DI-BTT permitted the clear identification of patients with β-thalassemia trait: DI-BTT was significantly higher in BTT patients and clearly separated them from NC and IDA patients.

In conclusion, the evaluation of CHr and MCVr distribution curves and the calculation of the DI-BTT can speed up the screening of IDA patients and β-thalassemia trait carriers, allowing the necessary tests to be performed to ensure the correct diagnosis and, thus, to begin the most appropriate treatment. Confirmation of the utility of this promising and low-cost method requires furthers confirmation on a larger number of cases.

## Figures and Tables

**Tab. 1:** Haematological parameters of β-thalassemia trait and Iron Deficiency Anemia

Parameters	β-Thal Trait * (n=72)	Iron Deficiency * (n=90)	P **
**RBC (x10^12^/L)**	5.81 ± 0.53	4.30 ± 0.69	<0.001
**MCV (fL)**	62.99 ± 5.98	77.95 ± 3.49	<0.001
**CHCM (g/dl)**	30.52 ± 1.88	33.51 ± 1.31	<0.001
**CHr (pg)**	21.44 ± 1.55	28.29 ± 1.42	<0.001
**MCVr (fL)**	82.06 ± 5.49	95.57 ± 3.4	<0.001
**CHCMr (g/dL)**	25.74 ± 1.73	29.68 ± 1.31	<0.001
**HbA_2_ (%)**	5.24 ± 0.94	2.88 ± 0.31	<0.001
**Ferritin (ng/mL)**	43.07 ± 21.78	10.50 ± 3.04	<0.001
**DI-BTT**	6.93 ± 1.64	3.71 ± 0.39	<0.001
**Serum Iron (μg/dL)**	74.00 ± 35.00	20.00 ± 9.00	<0.001
**Transferrin saturation (%)**	19.00 ± 8.00	4.76 ± 3.00	<0.001
**TIBC (μg/dL)**	340.00 ± 50.00	370.00 ± 83.00	0.06

* The results are shown as mean± standard deviation

** Statistical significance was calculated by chi square test or by Fisher exact test.

**Tab. 2:** Variation of reticulocyte indices between β-thalassemia trait (BTT), Iron Deficiency Anemia (IDA) and Normal Controls (NC)

	NC Vs BTT	P^1^*	NC Vs IDA	P^2^**	IDA Vs BTT	P^3^***
**Δ-CHr (pg)**	8.0	<0.001	1.15	<0.001	7.25	<0.001
**Δ-MCVr (fL)**	14.25	<0.001	0.74	<0.05	3.51	<0.001
**Δ-CHCMr (g/dL)**	4.91	<0.001	0.97	<0.001	3.94	<0.00

T- test analysis

* p1 (NC Vs BTT)

** p2 (NC Vs IDA)

*** p3 (BTT Vs IDA)

**Table 3:** Correlation between DI-BTT and β-thalassemia associated mutations

N	MCV (fL)	HbA_2_ (%)	DI-BTT	Mutation	% Mutation
**33**	60.09	5.86	7.682	CD39	54.10
**4**	59.33	5	7.555	IVS1.1	6.56
**6**	60.66	5.86	7.373	IVS2.1	9.84
**13**	62.75	5.05	7.134	IVS1.110	21.31
**8**	62.5	5.21	7.087	IVS2.745	13.11
**8**	67.24	4.49	6.866	IVS1.6	13.11

N = Patient number; MCV = Mean cellular volume); HbA_2_% = haemoglobin A_2_ percentage; DI-BTT = discriminant index β-Thalassemia Trait.
